# Nonmyocytes as electrophysiological contributors to cardiac excitation and conduction

**DOI:** 10.1152/ajpheart.00184.2023

**Published:** 2023-07-07

**Authors:** Ana Simon-Chica, Eike M. Wülfers, Peter Kohl

**Affiliations:** ^1^Novel Arrhythmogenic Mechanisms Program, Centro Nacional de Investigaciones Cardiovasculares, Madrid, Spain; ^2^Institute for Experimental Cardiovascular Medicine, University Heart Center Freiburg-Bad Krozingen, Faculty of Medicine, University of Freiburg, Freiburg, Germany; ^3^Department of Physics and Astronomy, Faculty of Sciences, Ghent University, Gent, Belgium

**Keywords:** cardiomyocyte, connexin-43, heterocellular coupling, nonmyocyte

## Abstract

Although cardiac action potential (AP) generation and propagation have traditionally been attributed exclusively to cardiomyocytes (CM), other cell types in the heart are also capable of forming electrically conducting junctions. Interactions between CM and nonmyocytes (NM) enable and modulate each other’s activity. This review provides an overview of the current understanding of heterocellular electrical communication in the heart. Although cardiac fibroblasts were initially thought to be electrical insulators, recent studies have demonstrated that they form functional electrical connections with CM in situ. Other NM, such as macrophages, have also been recognized as contributing to cardiac electrophysiology and arrhythmogenesis. Novel experimental tools have enabled the investigation of cell-specific activity patterns in native cardiac tissue, which is expected to yield exciting new insights into the development of novel or improved diagnostic and therapeutic strategies.

## INTRODUCTION

The heart is a biological pump whose mechanical activity is controlled by the generation and propagation of electrical signals, called action potentials (AP). Cardiac AP generation and propagation have traditionally been attributed exclusively to cardiomyocytes (CM). Although CM are undoubtedly the source of cardiac AP generation, they are not the only cell type capable of forming electrically conducting junctions in the heart. Thus, CM interact with nonmyocytes (NM), enabling and modulating each other’s activity. Recent flow cytometry and single-cell RNA sequencing (scRNA-seq) studies have rapidly expanded our appreciation of the cellular diversity, dynamism, and complexity of cardiac tissue organization. Nonetheless, when we consider electrophysiology, we still tend to focus on CM. This can be explained, in part at least, by technical limitations of conventional electrophysiology techniques to identify, isolate, and quantify electrical contributions from other cardiac cell populations. Pioneering studies on NM electrophysiology have focused on fibroblasts, cardiac NM that, in situ, show a particularly intimate spatial interrelation with CM. Cardiac fibroblasts were initially thought of as electrical insulators, even though they had long been known to electrically couple to CM in vitro. Building on advances in optogenetic and electrophysiological tools, functional CM-fibroblast coupling has been demonstrated in situ. Although fibroblasts were a perhaps obvious NM population to investigate first due to their abundance and roles in cardiac remodeling after injury, other NM such as macrophages have also been recognized as contributing to cardiac electrophysiology and arrhythmogenesis. The present review aims at summarizing current insight into direct and indirect effects of NM on cardiac electrophysiology, with a special focus on passive electrical (electrotonic) coupling between CM and NM in the heart.

## THE MYOCARDIUM: MUCH MORE THAN CM

Motivated by their experimentally established electrotonic coupling to CM in situ, this section introduces cardiac fibroblast and macrophage populations in physiological and pathophysiological conditions. The origin of these cells, their main physiological functions, and their basic electrophysiological properties are discussed. Although this section is mainly focused on fibroblasts and macrophages, it should be noted that the cellular composition of the heart is highly heterogeneous, with at least nine major cell types and 20 subtypes in human myocardium, including fibroblasts, immune cells (myeloid and lymphoid cells), mural (pericytes and vascular smooth muscle cells), and endothelial cells (1, 172). Other cell types such as adipocytes, neurons, or Schwann cells, and their roles in the patho-/physiology of the heart are receiving progressively more attention.

### Cardiac Fibroblasts—As We Know Them

Cardiac fibroblasts constitute one of the most abundant cell populations in the heart (2, 3). Independent of their location, fibroblasts are commonly referred to as mesenchymal cells that produce a diverse array of extracellular matrix (ECM) proteins, forming the connective tissue that supports essential aspects of organ structure and function (4). These cells were first described by the German pathologist Rudolf Virchow (5) as “spindle-shaped cells of the connective tissue.” Ziegler (6) later introduced the term “fibroblast” to describe cells that produce new connective tissue upon healing. The “-blast” suffix stems from “βλαστός” (*blastós*; Greek for germ, sprout, bud) and refers to morphological hallmarks of active synthesis of “fibrous” proteins. For all intents and purposes, this should be regarded as the definition of fibroblasts; alas, the majority of current research does not assess ECM component production when categorizing cardiac cell types.

In the heart, fibroblasts are present throughout all chambers, intermingling with CM. During physiological conditions, they contribute to tissue structural integrity by producing proteins that form the ECM, including collagen type I, one of the most abundant proteins in our bodies (7), as well as matrix metalloproteinases and tissue inhibitors of metalloproteinases, which in turn balance degradation of ECM components. It was not until the 1990s that cardiac fibroblast research experienced a massive shift from histoanatomical characterization toward functional assays, thanks to the discovery of fibroblast-mediated biochemical signaling and complex biophysical interactions with CM. This heterocellular cross talk influences tissue development and functional adaptation to physiological and pathophysiological challenges in many organs, including the heart. For more detail, the reader is referred to Refs. 8–13.

Cardiac fibroblasts originate from different progenitor populations. Epicardial and endocardial epithelial cells have been found to generate fibroblasts through epithelial-to-mesenchymal transition and endothelial-to-mesenchymal transition, respectively (14, 15). This was later confirmed using lineage tracing in mice (16). In addition, a fraction of fibroblasts has been shown to derive from circulating hematopoietic progenitors. At 7 days postmyocardial infarction, this proportion is increased to between a quarter (17) and two-thirds (18) of fibroblasts in postinjury scars. These discoveries highlight the wide diversity of cardiac fibroblast origins. Due to this heterogeneity, it may not be too surprising that specific markers of cardiac fibroblasts are still missing (13), as quite probably they are not “one” cell population.

Another interesting aspect of fibroblasts is their dynamic remodeling in response to external signals, including pathological conditions such as cardiac injury (leading to focal scars) or pressure/volume overload (leading to more diffuse fibrotic remodeling). Pathological remodeling involves changes in fibroblast phenotype, function, and subpopulation proportions. An example of this is tissue fibrosis, characterized by excess presence of ECM, where cardiac fibroblasts undergo a transition from fibroblasts to myofibroblasts (11). There are various methods to activate fibroblasts, with a prominent trigger being alterations in the mechanical and structural microenvironment, such as seen upon a loss of myocardial tissue integrity after injury (19). The consequences of fibroblast activation in the heart include increased proliferation and migration, augmented responsiveness to, and release of, signaling molecules, ECM deposition, and modifications in the expression of adhesion molecules such as integrins and their receptors (20). These processes are driven by the interplay of different fibroblast subpopulations whose functions may vary over time. In this regard, detailed analyses of scRNA-seq data from various organs have demonstrated high levels of fibroblast heterogeneity within and between tissues. As an illustration: in mouse, fewer than 20% of fibroblast-enriched genes overlap between heart, skeletal muscle, intestine, and bladder (21).

In addition to the relevance of fibroblasts in the aforementioned processes and functions, they also play direct roles in the electrophysiological behavior of myocardium. Although unable to generate AP (most of the cardiac NM, including fibroblasts, are not “excitable”), fibroblasts contain a collection of ion channels, pumps, and exchangers. These include voltage-activated, outwardly, or inwardly rectifying potassium (K^+^) channels (22–24), large conductance calcium (Ca^2+^)-activated K^+^ channels (25, 26), and voltage-dependent sodium (Na^+^) channels (24, 25, 27). Other ion flux pathways include proton permeable channels that may modulate the resting membrane potential (RMP) of fibroblasts based on their metabolic state (28), chloride channels (29), and stretch-activated ion channels (30, 31). The latter comprise Ca^2+^-activated nonspecific cationic transient receptor potential (TRP) channels [TRPC3, TRPC6, TRPM7, and TRPV4, reviewed in detail elsewhere (32, 33)]. Recent investigations also revealed the presence of Piezo1 in murine and human cardiac fibroblasts (34). Alterations in the expression and activity of this channel population have been linked to the pathophysiology of atrial fibrillation (26). Interestingly, changes in the mechanical stiffness of the ECM have been shown to affect Piezo1 ion channel dynamics in cardiac fibroblasts (35), highlighting the bidirectional cross talk between cardiac tissue components and properties.

Some of the ion channels introduced earlier, especially inwardly rectifying K^+^ channels, directly affect the RMP of fibroblasts. However, a high degree of heterogeneity in ion channel expression has been reported in different species under physiological and pathological conditions (24), and even across different heart chambers (36). The analysis of heterogeneities in expression must be done with care, considering preparations and experimental conditions [i.e., freshly isolated vs. cultured fibroblasts (22)]. In this context, RMP reported in the literature show large variability, from −15 ± 19 mV in rat-isolated fibroblasts (37) to −58 ± 3.9 mV in adult rat cultured (myo-)fibroblasts (23); cultured human atrial fibroblasts/myofibroblasts have an RMP of about −35 mV (24).

Even before any electrophysiological remodeling that tends to be caused by the effects of cell culture conditions, there are pronounced morphological differences between NM in vivo and in vitro. For example, in vivo, fibroblasts exhibit multiple elongated cytoplasmic processes and form large sheet-like extensions. In contrast, freshly isolated fibroblasts are initially nearly spherical cells containing the nucleus but few, if any, cytoplasmic processes and membrane invaginations. Although there is currently no data on the cell size distribution of fibroblasts in vivo, a total surface area of 1,500 μm^2^ or more has been estimated (38, 39). It is, therefore, reasonable to assume that fibroblast membrane capacitance in vivo exceeds levels observed in cells freshly isolated from healthy myocardium [typically with a surface area of ∼150–250 μm^2^ and a capacitance of 6–10 pF (22, 40)] by an order of magnitude. This is in keeping with the suggestion that fibroblast membrane resistances in situ are about an order of magnitude lower than in freshly isolated cells [0.5–1 vs. 1–10 GΩ (22, 41)].

In addition, atrial and ventricular fibroblasts are functionally different and there are chamber-specific responses during disease. Thus, in various heart disease models, the atria display a greater degree of fibrotic response, compared with the ventricles. For instance, studies of cultured atrial fibroblasts have shown that these cells exhibit a faster increase in cell surface area, a distinct morphology at confluence, and a higher expression of α-smooth muscle actin, compared with ventricular fibroblasts (36). Moreover, atrial tissue shows a higher density of myofibroblasts, compared with ventricular tissue, and this difference is even more pronounced in cases of congestive heart failure (36, 42).

### Cardiac Macrophages—As We Mostly Do Not Know Them

Macrophages, broadly defined by their canonical ability to phagocytose cell debris or other cells (from μακρός: *makrós* [Greek for large] and φαγεῖν: *phagein* [Greek for to eat]) and to stimulate other immune cells, were proposed in the 19th century to exclusively originate from monocytes (43). This concept was put into question when the same group observed that a specific population of macrophages in the spleen was maintained independently of monocytes (44), raising a critical question in the immunology field: where do tissue-resident macrophages come from?

Ground-breaking research in the last decade has demonstrated that tissue-resident macrophages are derived from embryonic progenitors. This finding holds true for several organs including lung, spleen, brain, and liver (45–48). It was observed that tissue-resident macrophages not only offer canonical phagocytosis-based protection but also possess vital organ-specific functions that contribute to the maintenance of tissue homeostasis [in view of dynamic adjustments to changes in physiological (and pathophysiological) demands, a better term would be tissue homeodynamics (49)]. In the heart, tissue-resident macrophages are the most abundant immune cell type under physiological conditions (50). With the development of additional sophisticated state-of-the-art tools, they are now recognized as a heterogeneous and ontogenically diverse population (51).

In terms of functionality, cardiac macrophages share similarities with microglia in brain. Initially, microglia was thought of as serving merely structural and/or trophic functions. However, accumulating evidence indicates that microglial cells interact with neurons in sophisticated ways that affect each other’s function, from the control of neuronal excitability to roles in brain repair and protection (52, 53). Functional data exploring cardiac macrophages in adult heart homeodynamics identified roles in coronary development (54), postnatal proliferation of CM (55), ECM remodeling (56) and, more recently, metabolic and morphological stability by clearing subcellular particles carrying cell fragments, for example, damaged mitochondria shed by CM (57). These exciting findings demonstrate specialized mechanisms, involving cardiac tissue macrophages that support individual CM integrity for the duration of postnatal life. This is particularly relevant for terminally differentiated cells that *1*) lack regenerative capacity, *2*) are exposed to high metabolic demands (57), and *3*) are required to keep their “identity” for the lifetime of the organism (this is true for CM and neurons of the central nervous system alike: they are “irreplaceable” as their demise has major implications for organ function and system viability). Besides their crucial role in supporting tissue homeodynamics, a broad variety of specialized functions have been attributed to cardiac macrophages in the diseased heart. During pathological conditions, the myocardium recruits a population of macrophages derived from circulating monocytes (expressing C-C chemokine receptor 2). This subpopulation plays crucial roles in replenishing the pool of cardiac macrophages, and shows proinflammatory and phagocytic activity (see Ref. 58 for a recent extensive review on the topic).

Like fibroblasts or CM, leukocytes express an array of ion channels. Initial studies that provided a first characterization of K^+^ channels in (peritoneal) macrophages date back to the 1980s when nonlinear current-voltage relationships were reported (59–61). Complex electrophysiological properties were described, where some macrophages had relatively depolarized RMP (−20 to −40 mV), whereas others exhibited more hyperpolarized RMP [−60 to −90 mV (59)]. These differences in RMP were interpreted as related to distinct functional states of activation. A deeper characterization indicated that voltage-dependent K^+^ channels in rat peritoneal and bone marrow-derived macrophages include the voltage-gated K^+^ channels K_v_1.3 and K_v_1.5, the inwardly rectifying K^+^ channel K_ir_2.1 (62), and the Ca^2+^-activated K^+^ channel K_Ca_3.1 (63). Similar to fibroblasts, the presence and extent of inwardly rectifying channels in macrophages promote a more negative RMP. These voltage-dependent K^+^ channels play a role in macrophage immunomodulatory responses, such as proliferation or activation (62, 64). K_v_1.3 can form heterotetramers with K_v_1.5, and changes in the stoichiometry of these ion channel proteins (i.e., changing the K_v_1.3 : K_v_1.5 ratio) can determine specific macrophage responses and biophysical properties (64, 65). Interestingly, anti-inflammatory treatment downregulates K_v_1.3 in heterometric channels, whereas inflammatory activation increases relative K_v_1.3 presence (66) further substantiating the link between electrophysiology and macrophage function.

More recently, murine cardiac resident macrophages were characterized using single-cell patch-clamp, RNA-seq, immunocytochemistry, and high-resolution three-dimensional (3-D) fluorescence/morphometric imaging (67). This work was conducted on freshly isolated macrophages, showing the following passive electrophysiological properties: an RMP of about −40 mV, a membrane resistance in the order of 1 GΩ, and a capacitance of just over 18 pF. Moreover, a comparison between freshly isolated, cultured, and in situ macrophages showed significant differences in shape and size: the in situ cell surface area was three times larger than that of freshly isolated cells, whereas cultured cells presented more elongated shapes and had a twofold larger surface area than immediately after isolation. From the analysis in terms of surface area, the authors reported that cultured macrophage capacitance was 1.9-fold higher than that of freshly isolated cells (34 pF vs. 18 pF), whereas their membrane resistance was lower by a similar factor. Consistent with findings from peritoneal or bone marrow-derived macrophages (64), several voltage-dependent K^+^ channels (K_v_1.3, K_v_1.5, and K_ir_2.1) were identified functionally.

Macrophages have the ability to sense their mechanical environment, which is thought to be critical for them to perform their housekeeping and immune functions. Stretch-activated ion channels such as Piezo1 and TRPV4 are ubiquitously expressed in the innate immune system. Piezo1 mediates Ca^2+^ influx in bone marrow-derived macrophages, subjected to cyclic changes in hydrostatic pressure which, in turn, modulates the expression of proinflammatory mediators such as interleukin (IL)-6, tumor necrosis factor-α, and chemokine ligand 2 (68). Similar to what has been reported for cardiac fibroblasts, Piezo1 expression in bone marrow-derived macrophages can be controlled by substrate stiffness (69). Resident cardiac macrophages sense mechanical stretch also through TRPV4 channels that regulate growth-factor expression (70). Other mechanosensitive ion channels have been identified at the mRNA level in resident cardiac macrophages (TRPV2, PIEZO1, TRPM7, and TRPP2), but are still awaiting characterization using functional assays (67).

Overall, fibroblasts and macrophages play important roles in the heart, contributing to its structural integrity, functional adaptation, and remodeling. Fibroblasts are present throughout the heart, intermingled between CM, maintaining the ECM, and contributing to tissue integrity. In response to disease, such as following myocardial infarction, they pheno-convert into activated myofibroblasts, contributing to tissue fibrosis. Macrophages, immune cells that phagocytose cell debris and stimulate other cardiac cells, are tissue-resident in the myocardium and contribute to tissue homeostasis, for example, in the context of coronary development, postnatal proliferation of CM, or ECM remodeling. During pathological conditions, additional monocyte-derived macrophages are recruited from the circulation and contribute to cardiac inflammation. Understanding their functions and interactions is crucial for studying cardiac physiology and pathology. Fibroblasts and macrophages express a variety of ion channels, which influence their electrophysiological properties. The specific expression and function of these ion channels may vary depending on the cell subtype, but also on species and experimental conditions.

## MECHANISMS OF CM-NM ELECTROTONIC COUPLING: FACTS AND GAPS

This section provides a critical perspective on the mechanisms of CM-NM electrotonic coupling. Electrical propagation through excitable CM is enabled by direct intercellular connections, formed by gap junctions (71), through which ions can flow directly from cell to cell. In working CM, gap junction channels are primarily located in a subdomain of the sarcolemma called the intercalated disk. Gap junctions are formed by a hexagonal arrangement of hemichannels (connexons, one in each of the abutting cell membranes) that are made up of six proteins [connexins (Cx) (72, 73)]. Cx37, Cx40, Cx43, and Cx45 have been observed in the heart, and differences between both cardiac regions and between working CM and pacemaking/conducting CM have been reported (74). The highly organized geometric arrangement and abundance of gap junctions within intercalated disks favors electrical propagation in the longitudinal CM direction, which supports synchronized excitation, excitation-contraction coupling, and effective cardiac contraction (75). NM also express Cx proteins, including Cx43, Cx45, and/or Cx40 at sites of homotypic (with other NM) and heterotypic (e.g., with CM) contact sites. The following sections explore the evidence for in vitro and in vivo heterocellular electrotonic interactions in the heart, and their possible functional implications for cardiac electrophysiology, based on experimental insight from biological (“wet-lab”) and computational (“dry-lab”) model systems.

### CM-Fibroblast Electrical Cross Talk

#### Insight from wet-lab models.

**Figure 1.**
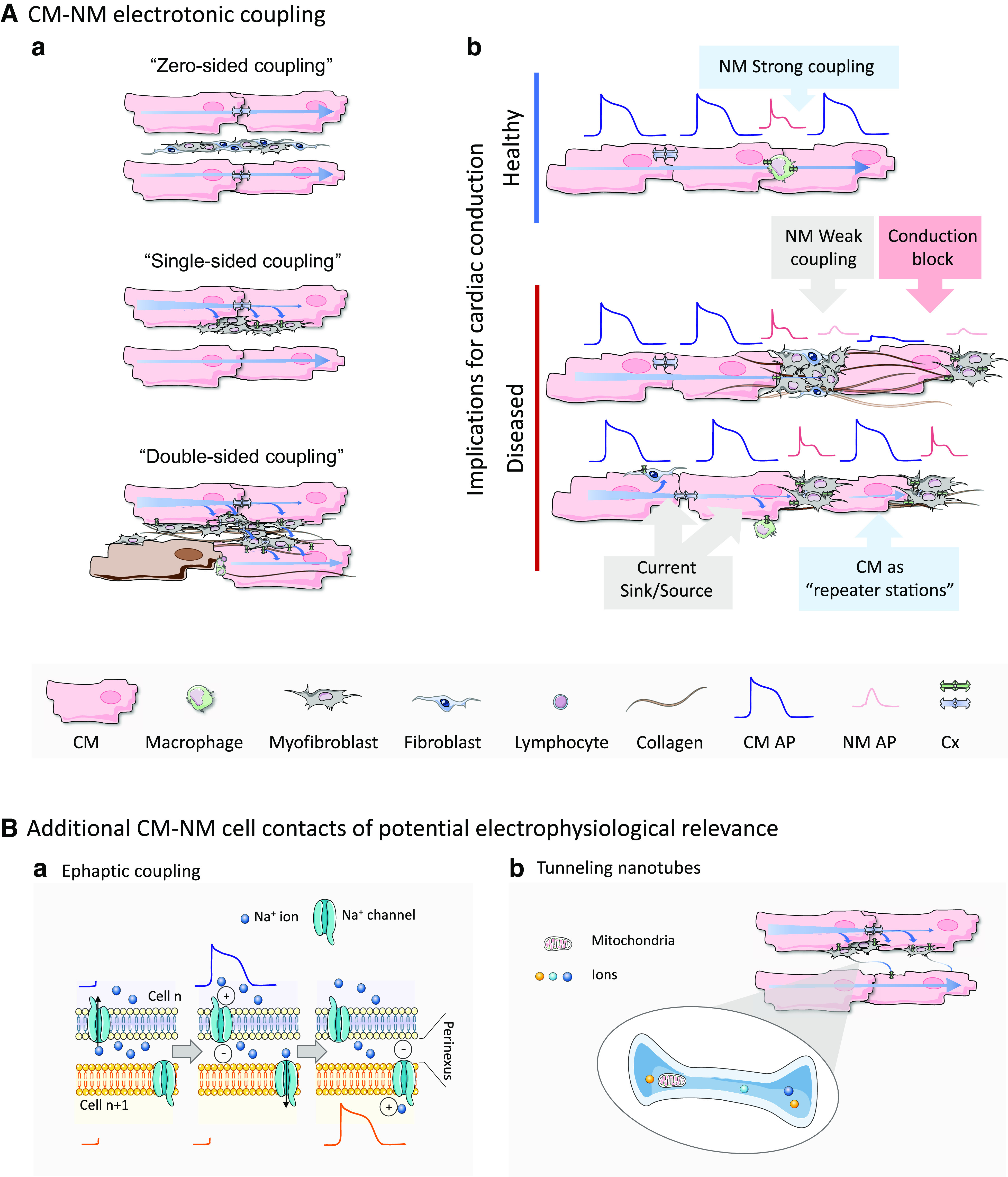
Cardiomyocyte-nonmyocyte (CM-NM) electrotonic coupling. *A*: conceptual contact-based interactions between CM and other NM via connexin (Cx) proteins (*a*) and their potential implications for cardiac conduction (*b*). Three basic scenarios are proposed in *a*: *1*) “zero-sided coupling” where there are no electrical connections between CM and NM, *2*) “single-sided coupling” where one or more NM are connected to one group of CM, and *3*) “double-sided coupling” where NM interlink CM that are not otherwise directly coupled electrically. In *b*, a schematic representation for strong and weak heterocellular coupling and their effects on electrical propagation is shown. In physiological conditions, NM may not have a prominent noticeable effect on cardiac conduction. In pathological settings, electrically coupled NM may passively bridge electrical conduction of CM action potentials (AP), or block conduction, if the “pseudo-AP” signal is too small to take downstream CM to the AP threshold. CM, interspersed between NM, may act as “repeater stations” by reconditioning the electrotonically conducted signal through active generation of an AP; this may allow bridging over distances that would otherwise fail to passively conduct excitation. *B*: additional CM-NM cell contacts of potential electrophysiological relevance. *a*: during ephaptic coupling, Na^+^ influx into a “prejunctional” cell may reduce the concentration of Na^+^ ions in a restricted volume of the extracellular cleft between cells. This then gives rise to a negative cleft potential, which may depolarize the “postjunctional” cell membrane. *b*: tunneling nanotubes connect two or more cells of homotypic or heterotypic nature, allowing direct exchange of ions, molecules, proteins, or even small organelles over long distances (micrometer range). Images were partly generated using Servier Medical Art, provided by Servier, licensed under a Creative Commons Attribution 3.0 unported license.

As early as the 1960s, Goshima and coauthors (76, 77) introduced the concept of cardiac heterocellular coupling, supported by the observation of effective synchronization of distant CM (≥150 μm), interlinked by multiple NM. The first circumstantial evidence for CM-fibroblast capacitative and electrotonic coupling in vivo involved the use of double-barreled microelectrodes that were inserted into subendocardial layers of spontaneously beating atria of rat (41) and frog (78). These studies showed that changes in the membrane potential of fibroblasts were correlated with the AP in neighboring CM. In 1992, Rook et al. (79) provided a detailed characterization of homo- and heterocellular gap junctions in vitro. They demonstrated that fibroblasts can connect distant CM via passive signal conduction, because of their high membrane resistance and low membrane capacitance. Using double whole cell patch-clamp experiments in cultures of neonatal rat CM and fibroblasts, they showed CM-like AP (although with reduced amplitude and slowed upstroke) in the electrotonically coupled fibroblast. Figure 1, *A* and *B* offers schematic representations of weak and strong heterocellular coupling and their effects on electrical propagation. Motivated by these findings, a novel, structured coculture system was implemented, which revealed that cardiac fibroblasts inserts can transmit electrical activation between two neonatal rat CM strands over distances of up to 300 µm (80). An additional study indicated that heterocellular gap junctional coupling influences cardiac electrical propagation by partially depolarizing CM, thus causing inactivation of Na^+^ channels and, accordingly, slowing conduction velocity (81). Neonatal rat CM monolayers cultured with fibroblasts from healthy and infarcted hearts established that cardiac injury enhances CM-fibroblasts interactions, as illustrated by fluorescence recovery after photobleaching (82). The presence of heterocellular electrotonic interactions between fibroblasts and CM was also demonstrated in 3-D cell culture models (83).

First evidence for heterocellular electrotonic coupling in native cardiac tissues involved electrophysiological, immunohistochemical, and dye transfer experiments in rabbit native atrial tissue (84). These observations, focused on the sinoatrial node (a tissue region rich in fibroblasts), highlighted that, although Cx40 is the predominant isoform found at contact points between fibroblasts, heterocellular coupling is dominated by Cx45 in the primary cardiac pacemaker region (84). Immunohistochemical findings from a sheep model on infarcted myocardium showed further that cardiac fibroblasts express Cx45 within hours of cardiac injury, whereas Cx43 expression in fibroblasts started to increase from 1 wk, and lasted at least for 1 mo, postinfarction (85); see also Ref. 86 for further details on the topic.

Following these enticing early studies and adopting an electrophysiological focus, further research explored heterocellular electrotonic coupling in situ, predominantly using animal models of injury, where scars or large areas composed of mostly NM were investigated. Walker et al. (87) used optical mapping of adult rabbit hearts to assess electrical activation of scar tissue 8 wk after coronary occlusion. They reported electrical propagation into the lesioned tissue and suggested an involvement of heterocellular coupling in the observed electrical invasion of large transmural infarcts. These findings were followed up by other studies showing cardiac excitation wave propagation, albeit attenuated, in the border zone of myocardial infarcts (88, 89). Mahoney et al. (90) used a myocardial cryoinjury mouse model where NM-specific conditional Cx43 knockout resulted in reduced wave propagation through scar tissue, lending further credence to an involvement of NM in electrical conduction. Of note, they used Cx43FSP1KO mice where Cx43 was knocked out in fibroblast-specific protein 1 (FSP1) positive cells. As FSP1 is expressed by multiple NM cell types, a more specific interpretation is difficult at the present time.

Another study used an inspiring new approach for treating cardiac conduction disorders using engineered electrical conduits (91). These were produced using paramagnetic beads coupled to surface-conjugated antibodies, which selectively bind to either neonatal rat CM or human stromal cells. The application of a linear magnetic field resulted in the formation of an elongated, structurally and functionally integrated, heterocellular tissue strand. In vitro studies demonstrated that the thus generated conduits can effectively synchronize the spontaneous electrical activity of disconnected regions of cardiac neonatal CM cultures. The approach was validated in vivo, where the ends of preformed CM/NM strands were attached to the right atrial and right ventricular epicardium. This procedure resulted in the formation of an atrioventricular (AV) conduction pathway, capable of supporting sequential chamber activation when the native intracardiac conduction system was inhibited by methacholine infusion. This indicates functional electrical coupling through heterotypic cell interactions in engineered cardiac tissue, potentially with exciting clinical implications for cardiac resynchronization therapies (91). One important benefit of incorporating CM, even if they do not form a continuous conductive pathway, is their ability to function as “repeater stations:” by reconditioning the electrotonically conducted impulse through active generation of an AP, they help to maintain effective electrical propagation throughout the graft’s input-to-output pathway (Fig. 1*B* and Fig. 3*F*) (92).

Despite this evidence, the in vivo presence and functional relevance of heterocellular coupling in the heart was viewed unfavorably for many decades. Reasons included not only the deviation from canonical textbook concepts, but valid concerns about the general applicability of in vitro findings that may be explained by overexpression of Cx43 in cultured NM, and the largely circumstantial nature of in vivo evidence. It was not until 2016 that Quinn et al. (93) unequivocally confirmed that NM can electrotonically couple to CM in situ. In this optical mapping study, they used an optogenetic approach that allowed measurement of cell-type-specific electrical activity, providing direct functional evidence of AP-like membrane potential changes in NM in left ventricular scar border zone tissue, 8 wk after cryoablation, in isolated Langendorff-perfused mouse hearts. These findings were subsequently corroborated by an independent study where electrical activity from CM and neighboring NM was measured simultaneously by confocal microscopy in the infarct border zone of Langendorff-perfused mouse hearts (94). These works illustrate how optical measurement of cardiac electrophysiology may serve as a powerful method for exploring cell-type-specific behavior in situ. This is particularly true for work using cell-type-specific expression of reporters, whether based on viral transduction or on breeding of recombinant mouse lines (95), to decipher the heterocellular nature of cardiac electrophysiology.

Although modern experimental methods have provided a deeper understanding of CM-NM coupling, it is still difficult to identify specific underlying mechanisms and causal chains of events involved in heterocellular electrical coupling. In this regard, computational modeling represents a powerful tool to assess the quantitative plausibility of hypotheses, complementing wet-lab experimentation. In particular, dry-lab models can help to explore interrelated structures and functions at multiple levels of organization (from subcellular to whole organ), and to do so dynamically (over time, such as during pathophysiological alterations) while controlling variables that are sometimes not accessible in experiments on biological models. Therefore, we next summarize major in silico findings and discuss the mechanistic insights into heterocellular electrotonic coupling afforded by them.

#### Insight from dry-lab models.

Following electrophysiological characterization of fibroblasts in vitro, mathematical models exploring their interactions with CM, and the arrhythmogenic implications thereof, emerged in the 1990s. In early models, fibroblasts were considered as an ohmic resistance connected to CM (the so-called “passive” model, cf. Refs. 41, 96–98). Although these passive models simplify the electrophysiological properties of fibroblasts to few core parameters (such as reversal potential, capacitance, membrane and coupling resistance), they were extensively used to provide important insight into plausible mechanisms and potential implications of heterocellular electrical coupling in multiscale computational models. Computer simulations demonstrated that CM-fibroblast coupling, through as few as 10–13 gap junctional channels (41) could lead to electrophysiologically relevant source-sink effects. The coupling would cause fibroblasts to slightly depolarize the RMP of CM, which could potentially accelerate pacemaker rate or even result in arrhythmogenesis (41, 78, 99, 100). In contrast, “active” fibroblast models include formulations for the different ion channels present in the cell [see *Cardiac Fibroblasts—As We Know Them*; (96, 101, 102)]. The term “active” is potentially misleading, though, as fibroblasts are of course modeled as nonexcitable cells. MacCannell et al. (96) showed that fibroblasts can affect currents involved in the repolarization of electrotonically coupled CM, resulting in AP duration changes. Computational modeling further suggests that the increase in CM RMP (partial depolarization), due to coupling to a less negative cell, can inactivate Na^+^ channels and delay Na^+^ channel recovery, extending postrepolarization refractoriness (103). In addition, CM-fibroblast coupling can give rise to Ca^2+^ alternans by affecting intracellular Ca^2+^ cycling (97).

Thus, computational studies, combined with in vitro and in vivo work, have demonstrated that the extent to which fibroblasts or myofibroblasts alter cardiac excitation and conduction is affected by *1*) the degree of heterocellular gap junctional coupling (associated with the number, conductance, and distribution of gap junctions connecting heterocellular cell pairs); *2*) the relative size and/or number of coupled cells; *3*) the RMP of coupled NM; and *4*) the specific cellular tissue architecture where (myo)fibroblasts intermingle with CM. Myofibroblasts, in particular, show upregulated expression of Cx43 in response to cardiac injury, potentially enhancing the effects of CM-NM heterocellular coupling (82).

Figure 1, *A* and *B* summarizes different possible interactions of CM and NM, and their implications for cardiac electrophysiology. Based on different configurations for CM-NM electrotonic coupling, three principal scenarios can be proposed (104, 105). First, “zero-sided coupling” refers to the case where there is no coupling between CM and NM (i.e., NM act as an electrical insulator, as is traditionally assumed and probably most often the case). The second is “single-sided coupling,” where one or more NM are connected to groups of CM that electrophysiologically act as a single unit. Here, NM form an additional, passive electrotonic load on CM. Third, “double-sided coupling” refers to connections where NM interlink groups of CM that are not otherwise directly coupled electrically. These interlinking connections are not limited to individual cells, as cardiac fibroblasts, for example, can be interconnected via homotypic gap junctions that support fibroblast-fibroblast electrotonic coupling in situ and in vitro. Double-sided coupling allows cardiac fibroblasts to form conducting pathways, as shown in cell cultures (76, 79, 80). This scenario may be relevant for cardiac electrophysiological behavior during postablation and postinfarction scar formation, which could have important therapeutic implications, for example, in the context of improving cardiac ablation by targeting NM to reduce the incidence of their homo- and heterotypic electrical connections across ablation lines: this could make cardiac ablation lines more durably insulating.

### CM-Macrophage Electrical Cross Talk

#### Insight from wet-lab models.

Cardiac macrophages have been proposed to mediate arrhythmogenesis through mechanisms such as production of cytokines linked to fibrosis formation and cardiac remodeling (106). Only a small number of studies have specifically addressed the effects of CM-macrophage heterocellular coupling on cardiac electrophysiology. Hulsmans et al. (107) first demonstrated that macrophages can be electrotonically coupled, via Cx43, to CM of the AV node of mice. Using optogenetic targeting of light-activated ion channels to allow selective depolarization of macrophages, or cell-type-specific Cx43 deletion in cardiac tissue-resident macrophages (in the absence of secondary inflammation associated with this intervention), they showed that resident macrophages can alter CM electrophysiology, affecting AV conduction in isolated mouse hearts. In addition, they used CD11b^DTR^ mice to deplete macrophages in an inducible way and observed progressive AV block in thus affected animals. In line with these findings, an independent group later reaffirmed that macrophages modulate CM electrophysiology via gap junctions in an in vitro myocardial infarction model (108). These authors identified K_Ca_3.1 as the most differentially expressed ion channel encoding gene (widely present in macrophages) in response to myocardial infarction. Inhibition of this channel attenuated postmyocardial infarction arrhythmogenesis in vivo (108).

#### Insight from dry-lab models.

Thus far, few studies have used in silico models of cardiac macrophages. To enable new insight into macrophage interactions with CM, a computational model has been developed incorporating passive and active electrophysiological properties of cardiac tissue-resident macrophages (67). This work showed that the effects of macrophages on CM electrophysiology are directly related to the RMP of coupled NM.

The most profound effects were predicted for macrophages expressing little to no inwardly rectifying K^+^ current, i.e., the macrophages with the least negative RMP. However, CM-macrophage electrotonic cross talk remains a relatively unexplored field, and major questions on the importance of this heterocellular coupling mechanism during physiological homeodynamics and pathological conditions are yet to be addressed. In general, one might expect CM-macrophage interactions to be significantly less stable (macrophages rove through the tissue to fulfill their “canonical” functions) than those of CM-fibroblast connections, but otherwise similar in terms of electrophysiological effects on CM.

To conclude, even under homeodynamic conditions, certain regions of the heart, such as the sinoatrial node, exhibit a particularly high density of fibroblasts, compared with other regions. In keeping with this, the first evidence for heterocellular electrotonic coupling in vivo had been found in rabbit sinoatrial node tissue (84). In general, though, heterocellular electrotonic coupling in the atria is less well explored than in the ventricles, where research has focused on lesioned myocardium (87–90, 93). With regard to other NM, it is worth noting that the healthy murine AV node is rich in resident macrophages [that can be electrotonically coupled to CM of the AV node (107)]. Comparative analyses of tissue-resident and recruited macrophages in cardiac lesions are yet to be provided. Overall, chamber- and disease-specific aspects of heterocellular electrotonic interactions of various NM with CM form an important target for further research.

### Other NM Cross Talk with CM?

Considering the heterocellular nature and vast range of NM in the myocardium, it is perhaps not surprising that gap junctions have been identified in many other cell types, including endothelial cells, pericytes, and adipocytes. Endothelial cells express a variety of connexins, including Cx40, Cx43, and Cx37, with Cx40 being most prevalent (109). A study by Narmoneva et al. (110) showed that endothelial cells play a role both in spatial organization, survival, and synchronization of neonatal CM in culture. In addition, endothelial cells were found to increase the expression of Cx43 in CM in a vascular endothelial growth factor-dependent manner, increasing both CM-CM and CM-endothelial cell coupling in vitro. There is also indirect evidence of gap junction-mediated intercellular communication between ventricular CM and microvascular endothelial cells in situ, potentially contributing to the regulation of arterial blood flow (111). Other roles of Cx43 are in vascular development, homeodynamics, and disease, including an involvement in endothelial and smooth muscle cell communication, regulation of blood flow and pressure, and modulation of inflammatory responses (112). In particular, Cx43 expression has been reported in pericytes at various developmental time points, including during early vessel formation (113). These data suggest the formation of gap junctions between pericytes and potentially between pericytes and endothelial cells. The thus-afforded intercellular communication seems to be associated with important functional implications: loss of Cx43 in pericyte progenitors compromizes embryonic blood vessel formation, whereas other connexin isoforms may have the ability to compensate for this loss in surviving animals. Pericytes are also believed to be involved in the “no reflow” phenomenon, which refers to a situation where a coronary artery is reopened after treatment of acute myocardial infarction, but tissue perfusion is not restored. It has been proposed that pericyte contraction, which occurs in response to reduced coronary perfusion, may cause capillary constriction, leading to no reflow, an effect thought to be mediated by the orphan receptor GPR39 in pericytes (114) and possibly spreading through cell-cell coupling. Targeting pericyte-endothelial communication with nonpharmacological or pharmacological interventions that relax pericytes could lead to a reduction in no-reflow, infarct size, and degree of ischemia (115).

Another NM type expressing gap junctions are adipocytes, as first demonstrated in epicardial fat of hamsters (116) and later confirmed in other mammals (117, 118). In a sheep myocardial infarction model, intramyocardial adipose tissue attenuated electrogram amplitude and exacerbated arrhythmogenicity (119). Although the authors did not assess whether adipocytes electrically couple to CM via gap junctions, they observed lateralization of Cx43 in CM close to adipose infiltrations. In vitro, Lin et al. (120) observed evidence for direct modulation of electrophysiological properties of CM by adipocytes, as rabbit left atrial CM, cocultured with adipocytes, exhibited a less negative RMP and longer AP durations. Interestingly, CM incubated with adipocyte-conditioned supernatant had longer AP duration as well, but unchanged RMP when compared with control CM. The RMP of isolated white adipocytes is approximately −30 mV, which could explain electrophysiological effects on CM if/when heterocellular electrotonic coupling is present (121). Further research is needed to explore how widespread heterocellular electrotonic coupling is in the heart (and other organs), and whether it can be modulated to decrease the propensity for arrhythmogenesis.

**Figure 2.**
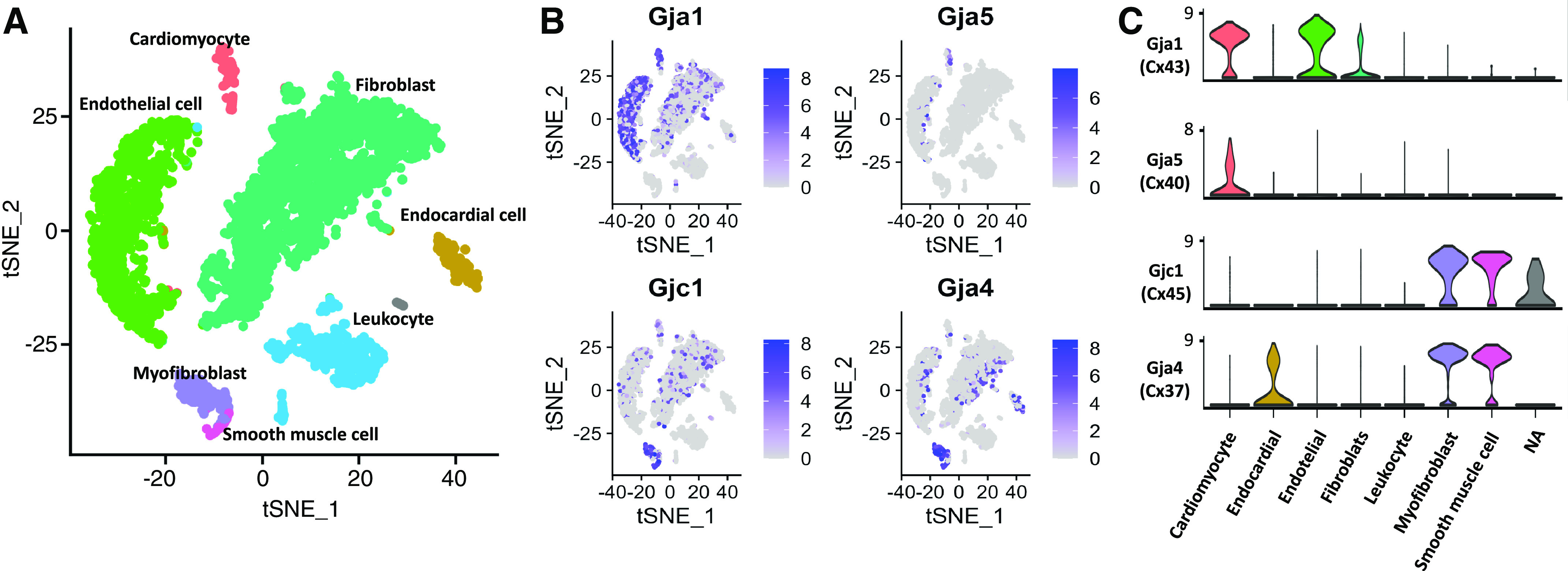
Connexin (Cx) gene expression in major cellular constituents of murine cardiac tissue. *A*: T-distributed stochastic neighbor-embedding (tSNE) plot where major cardiac cell populations are identified after unsupervised clustering. Each point depicts a single cell, colored according to cluster designation. *B*: Cx gene expression gradients identified within major cardiac cell populations. The points show cells positioned in tSNE space identically to *A*, with the more intense purple hue indicating higher relative Cx expression and gray signifying no expression. *C*: violin plots show expression of representative Cx genes in each cell type: Gja1, Cx43; Gja5, Cx40; Gjc1, Cx45; Gja4, Cx37.

Figure 2 summarizes Cx expression (Cx43, Cx40, Cx37, and Cx45) among different cardiac cell populations, reported in the Tabula Muris project, a compendium of single-cell transcriptomic data from *Mus musculus*, which comprises more than 100,000 cells from 20 organs and tissues (122). Of course, RNA-seq based assessment of gene expression is no proof of protein synthesis and/or localization to surface membranes or heterocellular contact points, and even observation of Cx proteins at heterocellular contact points is no proof of functional cell coupling, highlighting the need for functional assays, ideally in native myocardium (as Cx-expression patterns in culture are generally nonrepresentative of tissue in situ).

To conclude, the extent of electrical coupling of fibroblasts and CM in control conditions is ill explored; in contrast, in cardiac lesions fibroblasts/myofibroblasts can be electrically coupled to CM. For macrophages, the picture is “inverse,” in that published evidence suggests electrical coupling with CM in normal AV node tissue, whereas the extent of such coupling in lesions is ill explored. Connexins have been identified in other cell types within the myocardium, including endothelial cells and adipocytes. Further research is needed to understand the extent of heterocellular electrotonic coupling in the heart, its modulation, and its implications for normal cardiac function and arrhythmogenesis.

## LIMITATIONS AND FUTURE PERSPECTIVES

### Effects of Heterocellular Coupling Matter—But Are Hard to Study

Functional CM-NM heterocellular coupling has been confirmed in situ (93, 94, 107). Given the dynamic nature of cardiac tissue remodeling after injury, it is reasonable to expect changes in heterocellular interactions. Understanding interaction dynamics in vivo after injury may allow targeted control of CM-NM cross talk, potentially to improve outcomes of postinjury structural and functional remodeling (123). As an example, NM can be genetically modified to overexpress Cx43. This has been found to have an antiarrhythmic effect in vitro on CM monolayers (124) and in vivo, where injection of Cx43 expressing NM (125) or transduction of cells within left ventricular scar tissue using Cx43 viruses decreased the occurrence of tachy pacing-induced arrhythmias (126).

Direct measurement of NM electrophysiological properties in situ remains challenging, as NM that are electrotonically coupled to CM can exhibit membrane potential dynamics similar to CM (41, 79). Optogenetic approaches for reporting or steering electrophysiology in specific cell populations have been instrumental in developing current concepts, showing that heterocellular coupling appears to be more likely to affect cardiac electrophysiology in lesioned tissue, than in healthy myocardium. Transgenic mouse models offer the possibility of targeting optogenetic actuators and reporters to selected CM and NM populations (127). However, targeted delivery of viral vectors to hearts from nontransgenic animals, including human myocardium, remains a significant challenge in the field. Intravascular administration of vectors suffers from gene transfer into nontarget organs. Magnetic nanoparticles (or viruses conjugated to them) may be used to enhance local transduction efficiency of systemically applied vectors through transient application of strong magnetic fields (128, 129). Alternatively, ultrasound-assisted intracardiac injection has been proposed as a potential solution for local cardiac delivery while remaining “minimally invasive.” In terms of targeting specific cell populations, different viral vector systems in combination with selected capsid proteins show viral tropism toward specific cell types. For example, the adeno-associated viral (AAV) serotype AAV9 is credited with CM-preferential targeting (130). The effectiveness of AAV9 gene delivery can vary, depending on factors such as delivery method, dosage, or animal model used, and alternative AAV serotypes have been suggested for CM targeting in vitro (131). Cardiac NM have been classically targeted with envelope-pseudotyped lentiviral vectors, but use of ecotropic viruses can increase the efficiency and safety of viral delivery systems. For example, recent studies suggest that Moloney murine leukemia virus exhibits a high transduction rate of NM cells in embryonic mouse hearts (128, 129) compared to pseudotyped vectors, while not infecting other cardiac cell types (132). Cell-type-specific transgene expression can be increased using appropriate promoter systems, as previously described (133).

### Effects of Heterocellular Coupling Differ between Animal Models

**Figure 3.**
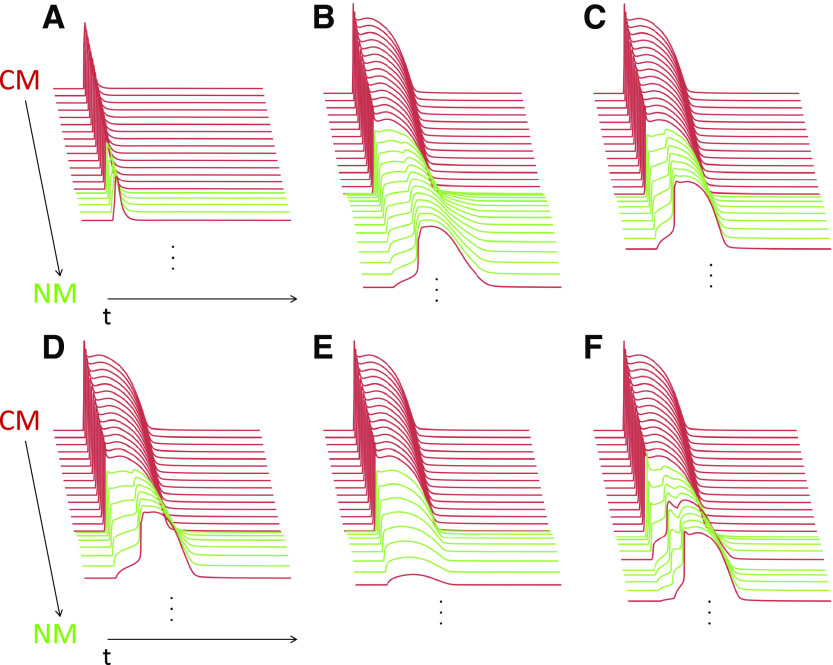
Computational modeling of passive action potential (AP) conduction through nonmyocyte (NM) inserts between groups of cardiomyocytes (CM). Fifteen CM (red), electrotonically connected to NM (green), with an AP triggered at the top. A short sharp murine AP can passively be conducted via a maximum of four NM and still excite a downstream CM (*A*) while the length of the NM insert can be up to 13 NM in the case of longer input signals such as AP of rabbit CM (*B*). NM hyperpolarization (from 0 to −12.1 mV in isolated cells) by K^+^-channel upregulation (*C*), or a 30% reduction in Cx43 coupling (*D*), roughly halve this maximum bridging distance (to 7 or 6 NM, respectively). CM, interspersed between NM, can act as “repeater stations” that recondition AP signal amplitude. This allows bridging over distances that would otherwise fail to passively conduct excitation: using the same conditions as in *D*, excitation of the distal CM fails after inserting 7 NM (*E*); this is countered by incorporation of one CM among eight NM (*F*). Please note the presence of bidirectional (antero- and retrograde) electrotonic effects in NM, seen as conducted depolarization waves that originate from excited CM at the middle or end of NM inserts.

The AP, controlled by a remarkably fine balance of ion fluxes, differs significantly between cardiac chambers and across species. Although the AP upstroke is preserved (mediated by the “fast” Na^+^ current Na_v_1.5), AP plateau and repolarization dynamics vary. In particular, ventricular CM of larger mammals (such as rabbit, pig, or human), and also of some smaller species (such as zebrafish), exhibit a pronounced plateau and comparatively long AP duration (200–500 ms), whereas small rodents have about an order of magnitude shorter ventricular AP with no pronounced plateau (20–50 ms; Fig. 3). These interspecies differences in cardiac electrophysiology may affect electrophysiological consequences of heterocellular coupling.

As ever, the choice of model system should depend on the specific research question at hand. In many cases, mice have added value because of the availability of transgenic models, including for optogenetic targeting of CM and NM. This is in addition to advantages related to the ease (and duration) of breeding, and the comparatively cheap maintenance costs, compared with rabbits or pigs. However, when investigating the distance over which NM may passively bridge electrical conduction of CM AP, shape and duration of the input-AP “upstream” of NM-inserts matter. As shown by computational modeling (Fig. 3), passive fibroblast-mediated conduction can occur over substantially longer distances when AP plateau levels are high and AP duration is long (compare Fig. 3, *A* and *B*), indicating that murine models may underestimate the electrophysiological importance of heterocellular electrotonic cross talk in the heart that may be present in human. As explained in *Cardiomyocyte-Fibroblast Electrical Cross Talk*, passive NM-mediated conduction depends on gap junctional conductance and RMP of coupled NM. Thus, a 30% reduction in gap junctional coupling between NM increases “pseudo-AP” signal attenuation in NM (compare Fig. 3, *B* and *C*), whereas hyperpolarization of electrotonically coupled NM (by upregulation of NM K^+^ channels) produces a similar conduction-curtailing effect on passive AP propagation in the model (Fig. 3, *B* and *D*).

As introduced in *Cardiomyocyte-Fibroblast Electrical Cross Talk*, CM interspersed between NM can act as “repeater stations,” allowing passive AP propagation over longer distances in tissue containing a mix of both cell types (Fig. 1*B* and Fig. 3, *E* and *F*). This scenario might be particularly relevant after ischemia-reperfusion injury, where NM in lesioned tissue tend to be interspersed with surviving CM. In postablation scars, whether by radio frequency (increases temperature), cryo (decreases temperature), pulsed electrical field (causes nonthermal membrane poration, potentially CM targeted), or surgical (mechanical cut) interventions, conduction block occurs over shorter distances, if all CM in the lesion are successfully eradicated. These simulations (compare Fig. 3, *A* and *B*) highlight the need to use models with AP durations that match those in human for the exploration of heterocellular interactions in translational, clinically relevant scenarios, such as postablation scars. A benefit of complementing experimental research with mathematical modeling is that this may help to translate insight between model systems, including projection from murine models to putative relevance for heterocellular coupling in human heart.

### Additional Heterocellular Contacts of Potential Electrophysiological Relevance

Although it is widely accepted that electrical propagation in working myocardium is enabled by Cx-based gap junctions (see mechanisms of cardiomyocyte-nonmyocyte electrotonic coupling: facts and gaps), other noncanonical forms of electrophysiologically relevant cell interaction have been described. For example, it has been suggested that dynamic changes in the ionic composition of the extracellular fluid in restricted spaces between cells (e.g., the perinexus that surrounds gap junctions) can contribute to heterocellular electrical cross talk, including effects on AP propagation. This mechanism of not gap junction-mediated coupling is termed ephaptic coupling and it is thought to arise as a consequence of Na^+^ influx into a “prejunctional” cell, which reduces the concentration of Na^+^ ions in the extracellular cleft between two cells. This then gives rise to a negative cleft potential (because of depletion of local positive charges in the perinexus) that may depolarize the “postjunctional” cell membrane (see Fig. 1*B*). This mechanism, initially proposed by Sperelakis and Mann (134) as an alternative to electrotonic coupling, was met with scepticism in as far as CM-mediated conduction in healthy myocardium is concerned as, via low-resistance Cx-mediated coupling, CM-coupling has a space constant that is understood to allow AP activation in cells much further “downstream” than the immediately neighboring CM. So, while the jury may still be out on the ultimate relevance of ephaptic coupling, it should be considered as a possible player, operating in tandem with gap junctions (135–137), especially under conditions of compromised electrotonic coupling, such as in myocardial ischemia (137, 138). Whether or not ephaptic coupling matters for electrophysiological cross talk between cardiac NM and CM remains to be elucidated.

Ephaptic coupling should not be confused with capacitive coupling, which has also been considered as a mechanism for heterocellular electrical interaction. When ion movements change the electrical potential of the cleft space, this may drive ephaptic coupling. In contrast, when the capacitive current of two juxtaposed membranes changes the electrical potential, interactions would be referred to as capacitive coupling. The latter has been considered for CM-fibroblast coupling in some detail, and based on mathematical modeling of effects, it was concluded to be unlikely to contribute to impulse conduction (39).

A third noncanonical mechanism of CM-NM coupling involves so-called tunneling nanotubes (Fig. 1*B*). These are long (µm range) and thin (Ø 50–200 nm) tubular conduits that can connect two or more cells of homotypic or heterotypic nature. Depending on configuration, tunneling nanotubes may allow direct exchange of ions, molecules, proteins, or even organelles (139). Since their discovery in vitro (140), tunneling nanotubes were found in a wide variety of cell types and tissues. A subset of tunneling nanotubes has been described as “closed ended” protrusions that require the expression of Cx at their tip to establish electrical coupling between cells (141, 142). He et al. (143) studied nanotube coupling between neonatal rat ventricular CM and fibroblast in vitro, revealing transfer of matter (ranging from Ca^2+^ ions to mitochondria) over long distances. They also showed indications of possible tunneling nanotube-mediated heterotypic cell contacts in adult mouse myocardium. The ample presence of heterocellular tunneling nanotubes was subsequently confirmed in murine left ventricular scar borders, using 3-D electron tomography (93), as these nanostructures are extremely difficult to detect in two-dimensional tissue sections (144). Consistent with these findings, the number of tunneling nanotubes between CM and fibroblasts was found to be significantly increased in vitro under ischemic coculture conditions (145). At the same time, the functional relevance of tunneling nanotubes between CM and NM in vivo is ill explored. Further insight will need to combine 3-D nanoscopic reconstruction and in vivo electrophysiology measurements, for example, using newly emerging optical approaches (146, 147). In summary, noncanonical forms of heterocellular coupling and their functional relevance form an exciting and underinvestigated facet of structure and function of the heterocellular heart.

### Indirect Heterocellular Cross Talk and Potential Electrophysiological Relevance

NM communication with other cardiac cells via paracrine signaling allows for contact-free cross talk that may affect cardiac electrophysiology. Among the first paracrine effectors identified in the context of cardiac fibrosis are angiotensin II and the cytokine transforming growth factor beta (TGF-β). Angiotensin II induces CM swelling, which has direct arrhythmogenic effects and is known to impair gap junctional coupling in rat ventricular CM (148). TGF-β is induced in the context of cardiac fibrosis and has been shown to promote myofibroblast differentiation and ECM production, contributing to the development of fibrosis (149). In addition to structural remodeling, paracrine factors released by NM can also affect ion channel and gap junctional expression. For example, fibroblast-conditioned medium causes a dose-dependent decrease in rat neonatal CM monolayer conduction velocity, prolongation of AP duration, depolarization of RMP, and decreased upstroke velocity (150). Similar observations were made with adult rat CM and adult mouse cardiac fibroblast-conditioned medium, showing a decrease in peak transient outward K^+^ current, lengthening of AP duration, and a decrease in Na^+^ channel current (151). Further studies are needed to expand on these findings in CM with inherently longer AP duration.

Activation of the immune system via paracrine mechanisms is part and parcel of homeodynamics and of the development of cardiovascular diseases, including those that lead to cardiac arrhythmias (152). A causal relationship between inflammation and arrhythmia has been proposed, see Ref. 153 for an extensive review of the topic. Cardiac macrophages induce electrical and structural remodeling, in part by releasing cytokines and chemokines. Indeed, investigating the complex effects of cytokines, released by leukocytes, on CM electrophysiology and conduction is starting to emerge as an exciting focus of research. In the setting of atrial fibrillation, recruitment of macrophages has been observed in left and right atria (154–156). These macrophages appear to have proinflammatory effects, releasing IL-1β that increases L-type Ca^2+^ current density in neonatal mouse ventricular CM (157), affecting excitation-contraction coupling and AP duration. Furthermore, in a mouse model of diabetes mellitus it has been demonstrated that hyperglycemia stimulates Toll-like receptor 2 and NLRP3 inflammasome in cardiac macrophages recruited from the circulation. This, in turn, triggers the release of IL-1β, potentially contributing to the development of ventricular arrhythmias by prolonging the ventricular AP and decreasing transient outward K^+^ channel current (158). Sun et al. (159) demonstrated that depletion of proinflammatory macrophages (lipopolysaccharide-induced macrophages) with clodronate liposomes reduces atrial fibrillation inducibility, indicating that macrophages may indeed have a causal role in atrial arrhythmia development or sustenance. Complementary to this study, Sugita et al. (160) showed that cardiac macrophages are essential for maintaining cardiac impulse conduction in a right-heart pressure overload mouse model. The described mechanism involves macrophage-derived amphiregulin as a key mediator controlling Cx43 phosphorylation and translocation in CM. Depletion of macrophages in this model resulted in advanced heart block and lethal cardiac arrest, in keeping with earlier observations (107). Other inflammatory mediators, such as IL-6, were shown to downregulate cardiac Cx levels, including Cx43 (161). The potential benefit of controlling sterile inflammation in the heart is further supported by data obtained in patients, in whom the administration of monoclonal antibodies against IL-1 β has been shown to decrease the rate of adverse cardiovascular events, compared with conventional treatment (162). Recently, Hulsmans et al. (173) have shown large-scale expansion of recruited macrophages in the atria of patients and mice during atrial fibrillation, as evidenced by scRNA-seq. They developed a macrophage-targeted therapy in mice, specifically deleting either Ccr2 (a chemokine receptor expressed in recruited macrophages) or Spp1 (the most upregulated macrophage-derived signal) to demonstrate a potential causal contribution to atrial fibrillation inducibility.

Innate immune cells not only affect CM electrophysiology by releasing inflammatory mediators, but there also is complex and dynamic cross talk between NM that appear to shape the onset and development of cardiovascular diseases. Paracrine factors released by macrophages including fibroblast growth factors, platelet-derived growth factors, vascular endothelial growth factors, IL-6, IL-13, and TGF-β1 (the most active profibrotic agent) activate fibroblast phenoconversion into myofibroblasts, resulting in increased collagen deposition and transcription of profibrotic genes (163). Some macrophage subpopulations (due to their innate predisposition or localization) may be more prone to activate fibroblasts and drive fibrosis than others. For instance, depletion of cardiac tissue-resident macrophages enhanced fibrosis and blunted angiogenesis in response to cardiac pressure overload (164). However, depletion of macrophages during different phases of tissue repair (injury vs. reparative) can have substantially differing effects on fibrotic outcome (165). Of course, the impression that fibroblasts are inactive, unless activated by other NM, would not be correct either (166). Furthermore, fibroblasts affect phenotype and function of other cardiac cells in different ways. For instance, in vitro contracting fibroblasts can generate dynamic mechanical gradients in the environment that influence and attract migrating macrophages over longer distances than chemotactic gradients (167).

Finally, mesenchymal stem cells (MSC) have received much attention in cardiovascular research, because of their potential therapeutic applications in treating various cardiovascular diseases. MSC are considered a class of adult pluripotent stem cells with multiple differentiation potentials, and they have been shown to have supportive roles as stromal cells. Some studies found that MSC can modulate CM ion channel/pump activity, for instance, enhancing sarcoplasmic reticulum Ca^2+^-ATPase function through paracrine actions (168). In the same vein, Askar et al. (169) found that MSC-conditioned medium prolonged AP duration of neonatal rat CM through paracrine signaling. In addition, there is evidence that MSC remodel in disease and change secretion of inflammatory cytokines. Among the MSC, secretome, IL-1β, and IL-6 are suspected to modulate inflammation and to contribute to the formation of arrhythmogenic substrates. The electrophysiological effects of MSC appear to be variable, depending on species and experimental conditions, so further research is needed to explore their paracrine effects on cardiac electrophysiology. For further information please see recent reviews (170, 171).

## CONCLUDING REMARKS

To conclude, there has been an expansion in recent years of novel experimental tools that can be used to investigate cell-specific electrical activity patterns, and to explore how those are linked to heterocellular coupling in native cardiac tissue. These tools are predicted to yield exciting new insight into the electrical integration across the diverse cell types that constitute the heart: new multi-omics approaches will allow us to take a fresh look at what determines a cell type; we will improve our understanding of regional differences in various parts of the heart and abutting vessels (such as pulmonary veins, a crucial driver of atrial fibrillation in patients); and finally, we expect new insight into dynamic aspects of development, aging, remodeling, and therapy (all of which are based on more than just one cell type in the heart). The cellular diversity, dynamism, and complexity of cardiac structural and functional organization are also being incorporated into computational models, with the view of aiding the interpretation of basic science and the projection toward translational relevance. An improved understanding of the nature and dynamics of heterocellular electrical communication in the heart requires insight into cell identities, their interactions, and the implications for development of novel/improved preventive, diagnostic, and therapeutic strategies.

## GRANTS

A.S.-C. received financial support via a “la Caixa” Foundation under ID 100010434 and PhD Fellowship LCF/BQ/DR19/11740029.

## DISCLOSURES

A.S.-C., E.M.W., and P.K. are members of the Deutsche Forschungsgemeinschaft (DFG)-funded Collaborative Research Center CRC1425 Project 422681845. None of the other authors has any conflicts of interest, financial or otherwise, to disclose. 

## AUTHOR CONTRIBUTIONS

A.S.-C. and P.K. conceived and designed research; A.S.-C. and E.M.W. prepared figures; A.S.-C. and P.K. drafted manuscript; A.S.-C., E.M.W., and P.K. edited and revised manuscript; A.S.-C., E.M.W., and P.K. approved final version of manuscript.
